# Childcare centre attendance and health, growth, and development among children aged 0–3 years in low- and middle-income countries: A systematic review

**DOI:** 10.7189/jogh.14.04028

**Published:** 2024-02-23

**Authors:** Farah Behbehani, Alysse J Kowalski, Helina Selam, Eileen Dombrowski, Maureen M Black

**Affiliations:** 1 Department of Public Health Practice, Kuwait University College of Public Health, Kuwait City, Kuwait; 2 Department of International Health, Johns Hopkins Bloomberg School of Public Health, Baltimore, Maryland, USA; 3 Department of Pediatrics, University of Maryland School of Medicine, Baltimore, Maryland, USA; 4 Vermont Oxford Network, Burlington, Vermont, USA; 5 School-to-School International, Pacifica, California, USA; 6 RTI International, Research Triangle Park, North Carolina, USA

## Abstract

**Background:**

Lack of childcare for children aged 0–3 years has emerged as a global crisis, accentuated by women’s increasing workforce participation and recognition that young children require nurturing care. Through this systematic review, we sought to examine associations between childcare centre attendance in low- and middle-income countries (LMICs) and children’s health, growth, and development, and to generate childcare centre programmatic and research recommendations for children aged 0–3 years.

**Methods:**

We systematically searched PsycINFO, MEDLINE, PubMed, and Cochrane for articles on centre-based childcare for children aged 0–3 years in LMICs, published between 2000 and 2021 in English (or which were translated into English). We excluded articles on specialised subgroups or interventions. We imported the retrieved articles into Covidence for review and assessed them for bias using the National Institutes of Health (NIH) quality assessment tool.

**Results:**

Twenty-two articles (24 studies) met the inclusion criteria, encompassing 36 927 children from 10 countries across Mexico and South America (n = 12), Africa (n = 5), and Asia (n = 5). Outcomes included health (n = 12), growth/nutrition (n = 6), and development (n = 6). Study quality assessments were low; 41% exceeded 50% of quality criteria and 45% adjusted for confounders. Associations between childcare attendance and outcome measures were primarily negative for health (n/N = 7/12) and positive for growth/nutrition (n/N = 5/6) and development (n/N = 4/6). Childcare centre programmatic recommendations for children aged 0–3 years included: age-specific policies; program quality, including safety, hygiene, nutrition, and curriculum; access and affordability; parent engagement; financial support; and workforce development. Research recommendations included: study design, including enrolment age, frequency, duration, childcare type, home and childcare sociodemographic and cultural environments, child and caregiver outcomes, and analytical approaches; longitudinal studies; and implementation research.

**Conclusions:**

Rigorous primary research in global childcare for young children is urgently needed. Policies, programmes, and investments in high-quality childcare can promote nurturing care for young children, enabling mothers to participate in the workforce.

**Registration:**

PROSPERO: CRD42018105576.

The lack of childcare globally represents a critical threat to the health and well-being of young children, to women’s employment, and to global economies. With women’s increasing participation in the workforce, demands for childcare have increased significantly. The World Bank estimated that over 40% of children under five years of age globally (approximately 350 million children) have unmet childcare needs [1]. Although pre-primary education has increased globally [2], most programmes serve children aged 3–5 years, with few services for those under three years old.

The period from birth to three years of age is characterised by rapid acceleration in growth and development, unique nutrition and feeding requirements, and vulnerability to infections as protective immunities develop [3]. During this period, children are highly sensitive to caregiving interactions and are forming the building blocks for lifelong health and well-being [4]. Based on the Nurturing Care Framework, young children need adequate nutrition and health care in the context of responsive nurturing environments, with opportunities for play and learning, as well as protection from adversities [5,6]. Recognition of the formative aspects of early childhood, coupled with women’s increasing participation in the workforce, have accelerated demands for childcare for children under three years of age.

There are few global guidelines for early childcare; in fact, the relationship between childcare attendance and young children’s health, growth, and development in low- and middle-income countries (LMICs) has generally been understudied, particularly among children under three years of age and in low-income settings [7]. Two reviews conducted in 2012 and 2014 studied children under six years of age in LMICs and found beneficial effects of childcare over home-based care on children’s motor, language, and psychosocial skills, but also higher rates of aggression, with inconsistent findings on children’s health and growth [8,9]. However, they did not differentiate findings for children under three years of age, and most of the studies they included were conducted in pre-primary schools serving children aged 3–5 years.

Our research question was: How does childcare attendance in LMICs affect the health, growth, and development of children under three years of age? To answer this, we aimed to conduct a systematic review of associations between childcare centre attendance in LMICs and the health, growth, and development of children under three years of age, and to generate childcare centre programmatic and research recommendations to provide nurturing care for children aged 0–3 years.

## METHODS

We conducted this systematic review per the PRISMA guidelines [10] and registered the protocol in the PROSPERO database (registration number: CRD42018105576).

### Search strategy

We conducted structured searches in PubMed, Embase, Cochrane Central Register of Controlled Trials, PsycINFO, and Education Resources Information Center (ERIC) databases on 9 October 2021, restricted to studies published over the past two decades (2000–21) in English or with English translation. Eligible references had at least one term from each of three concepts: childcare centre terms, including childcare, early childhood care, preschool, and nursery school; children under age five years; and LMICs, as defined by the World Bank [11]. The search was limited to childcare centres exclusively and did not include informal care provided by family or friends. Outcome measures were broad to capture relations between childcare attendance and children’s health, growth, and development. We adjusted search terms for each database, incorporating both subject headings and keywords.

### Eligibility criteria

We included studies on children aged 0–3 years attending centre-based childcare in LMICs, compared to children not attending childcare (usually in home care) which looked at a health, growth, or development outcome, even if they did not primarily focus on childcare attendance. We excluded studies solely focused on specialised subgroups (e.g. additional or special needs), outcomes (e.g. oral health), or interventions (e.g. nutrition supplementation).

### Data extraction and synthesis

We imported the retrieved articles into Covidence (Veritas Health Innovation, Melbourne, Australia) [12] and removed duplicates. To determine eligibility, two reviewers (FB, AK, HS, ED, or MB) independently screened the titles and abstracts for inclusion, resolving conflicts by consensus and team consultation. Two reviewers then read the full text of identified articles to select the final set of articles, resolving conflicts by consensus and team consultation. Finally, two reviewers independently read the full text of the final set of articles and extracted pre-specified information into a custom Covidence template that had been pilot-tested on five articles. The template included: study characteristics (authors, publication year, study design, country), study sample characteristics (sample size, child age, recruitment, inclusion/exclusion criteria, baseline differences between comparison groups), childcare centre characteristics (duration of attendance, age of entry), and outcome measurement and findings (outcome measure, report vs direct measurement, measure of association). A third reviewer conducted a consensus review, consulting a team if discrepancies could not be resolved. We reached out to authors if data within the article unclear. If an article included multiple outcomes, we considered each as a separate study. We grouped the studies by the type of outcome measure and summarised the findings in a narrative review, noting whether the associations between childcare attendance and outcomes were positive, negative, or if there was no difference. We did not perform a meta-analysis due to the heterogeneity of included studies.

### Quality assessment

We assessed the articles for bias using the National Institutes of Health (NIH) National Heart, Lung, and Blood Institute Study Quality Assessment Tool for Observational Cohort and Cross-Sectional Studies [13]. The tool includes 14 items that address concepts key to internal validity, including research question, study population, recruitment and eligibility, sample size justification, exposure assessed prior to outcome measurement, sufficient timeframe for an effect, levels of exposure, exposure measurement and assessment, repeated exposure assessment, outcome measures, blinding of outcome assessors, follow-up rate, and statistical analyses. Items are rated as ‘Yes,’ ‘No,’ ‘Cannot determine,’ ‘Not reported,’ or ‘Not applicable.’ This assessment was done by two reviewers (FB, AK, HS, or MB), followed by a third reviewer who resolved any disagreements through consensus. Risk of bias was summarised by tallying the number of criteria met for each article within the 14 quality criteria. We retained all articles for the narrative review, regardless of quality, due to their low overall number.

### Childcare centre programmatic and research recommendations

Childcare centre programmatic and research recommendations for children aged 0–3 years were informed by the systematic review, augmented by childcare criteria for children under three years of age from Zero-To-Three, a non-governmental organisation that provides programmes, training, resources, and policy solutions related to children aged 0–3 years [14]; publications that address global childcare quality and policies [1,15,16]; and the health, nutrition, and developmental needs of infants and young children [3,5,17]. Following the data extraction and quality assessment, we integrated the findings with the criteria for young children and generated childcare centre programmatic and research recommendations for children aged 0–3 years.

## RESULTS

**Figure 1:**
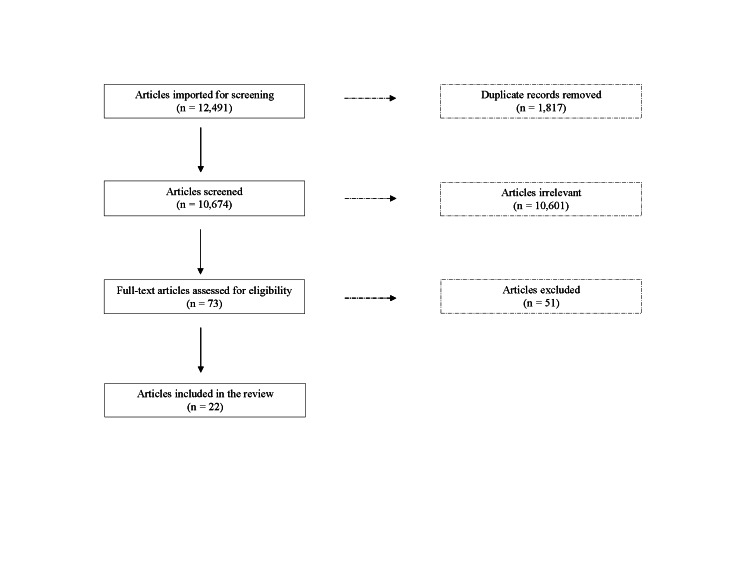
Flowchart of study inclusion process according to PRISMA guideline.

The initial search retrieved 12 491 articles; we removed 1817 through deduplication. We screened the titles and abstracts of the remaining 10 674 article, retaining 73 for full-text review. We excluded a further 51 articles due to having an ineligible study population, lacking a comparison group, or focusing on specialised outcomes. We finally included 22 articles; two reported on multiple outcomes, resulting in a total of 24 studies (**Figure 1** and **Table 1**). The included articles reported on studies conducted in Mexico or South America (n = 12), Africa (n = 5), and Asia (n = 5), with primary outcomes of health (n = 12), growth or nutrition (n = 6), and child development (including adult-child interaction and physical activity) (n = 6).

**Table 1:** Summary of included studies

**Study (year), country**	**Outcome**	**Design**	Age range	Sample size (% CC)	Outcome assessment	Recruit CC/COMP; Group Diff	Findings	CC effect
**Health**								
Asoegwu et al. (2013) [19], Nigeria	OME	X-sect	6–24 mo	152 (42.1)	MV	CC/HC; Yes	OME: CC = COMP	ND
							OME: early CC enrolment	
							OME: longer CC attendance	
Satoh et al. (2021) [18], Vietnam	OME	X-sect	4–11 mo 14–23 mo	274 (23.7)	MV	HC; NR	OME: CC > COMP	NEG
Gurgel et al. (2005) [20], Brazil	Intestinal parasitosis	X-sect	1–5 y	468 (46.8)	MV	CC/Comm; Yes	Intestinal parasitosis: CC > COMP	NEG
Clark et al. (2021) [22], Kenya*	Dia, health, fever, cough	X-sect; Qual	1–3 y	1337 (37.3); 31 interview	Survey and interview guide, PR	2015 NUHDSS; NR	Dia: CC < COMP	POS
							Fever, cough: CC = COMP	
							Health (PR employed mothers): CC > COMP	
Oliveira et al. (2019) [21], Brazil*	Dia	X-sect	12 mo	4018 (11.7)	Survey, PR	2015 PBC; Yes	Dia: CC > COMP	NEG
							Dia: Recent CC entry	
							Dia: private CC > COMP	
							Dia: public CC = COMP	
Hussen et al. (2020) [24] Ethiopia	*Streptococcus* pneum (nasopharynx)	X-sect	3–59 mo	413 (32.4)	MV	HC; NR	Nasopharyngeal *Streptococcus* pneum: CC > COMP	ND
Larenas-Linnemann et al. (2020) [28], Mexico	Atopic and nonatopic wheezing	X-sect	<12 mo	703 (17.6)	Survey, PR	Comm/HC; NR	Atopic wheezing: CC = COMP	ND
							Nonatopic wheezing: CC = COMP	
Mohamed et al. (2021) [25], Vietnam	*Streptococcus* pneum (conjunctiva)	X-sect	4–23 mo	698 (23.0)	MV	Comm records; NR	Conjunctival *Streptococcus* pneum: CC > COMP	NEG
Nguyen et al. (2019) [26], Vietnam	Pneum	X-sect	2–59 mo	4206 (50.0)	MV	Hosp admit; NR	Pneum-related adverse outcomes: CC < COMP	POS
Oliveira et al. (2019) [21], Brazil*	Infectious morbidities (respiratory symptoms, URI, LRI, flu/cold)	X-sect	12 mo	4018 (11.8)	Survey, PR	2015 PBC; Yes	NS respiratory symptoms: CC > COMP	NEG
							URI: CC > COMP	
							LRI: CC > COMP	
							Flu/cold: CC = COMP	
Vieira et al. (2006) [23], Brazil	*Streptococcus pyogenes* (oropharynx)	X-sect	3 mo to 8 y	200 (50.0)	MV	CC/HC; Yes	Oropharyngeal *Streptococcus pyogenes*: Sao Paulo CC > COMP, Porto Velho CC = COMP	NEG
Wong-Chew et al. (2017) [27], Mexico	Pneum	X-sect	1 mo to 5 y	1404 (7.1)	MV	Hosp admit; NR	Severe pneum: CC > COMP	NEG
**Growth**								
Madiba et al. (2019) [29], South Africa	Stunting, UW, wasting	X-sect	12-60 mo	1254 (39.5)	Anthro	HC; No	Stunting: CC < COMP	POS
							UW: CC < COMP	
							Wasting: CC = COMP	
Poudel et al. (2004) [30], Nepal	Stunting, UW	X-sect	1–5 y	46 (50.0)	Anthro	CC (municipal, NGO)/Comm; No	Stunting: COMP > CC	POS
							UW: CC = COMP	
							Stunting (NGO): COMP > CC	
							Stunting (Municipal), UW: CC = COMP	
da Silva et al. (2000) [31], Brazil	Stunting, UW, wasting	X-sect	<6 y	10 667 (13.0)	Anthro	1989 NSHN; Yes	Stunting: CC < COMP	POS
							UW: CC < COMP	
							Wasting: CC < COMP	
Silva et al. (2000) [32], Brazil	Malnutrition	X-sect	0–6 y	2122 (21.0)	Anthro	CC/Vax campaign; Yes	% malnourished: CC < COMP	POS
							Malnutrition: CC attendance >1 y < attendance ≤1 y	
Allel et al. (2020) [34], Chile	BMI, OW	Cohort	12–24 mo	1273 (35.0)	Anthro	2010 Chilean ECLS; Yes	BMI: CC < COMP	POS
							OW: CC = COMP	
Asekun-Olarinmoye et al. (2011) [33], Nigeria	EBF, BF duration	X-sect	0–2 y	500 (50.0)	Survey, PR/TR	CC/Comm; Yes	EBF: COMP > CC	NEG
							BF: duration 0–12 m COMP > CC	
**Development**								
Atay et al. (2015) [37], Turkey	QOL	X-sect	2–4 y	168 (31.0)	Paediatric QOL, PR/TR	HC; Yes	Psychosocial health: CC > COMP	POS
							Physical health, psychosocial, social, & emotional: CC > COMP	
Clark et al. (2021) [22], Kenya*	Neuro-dev	X-sect; Qual	1–3 y	1337 (37.0); 31 interview	Ages and stages, DM/PR; Interview guide, PR	2015 NUHDSS; NR	Cognitive: CC > COMP	POS
							Qualitative: childcare benefits cognitive, social, and emotional development. Childcare ensures future educational success and escape from poverty	
Leão et al. (2021) [36], Brazil	Neuro-dev	Cohort	21–27 mo	3870 (33.0)	INTER-GROWTH-21^st^, DM	2015 PBC; Yes	Cognitive: CC > COMP	POS
							Fine motor, gross motor, language: CC = COMP	
Lordelo et al. (2007) [35], Brazil	Neuro-dev	Long	1–3 y	32 (50.0)	Bayley/ WPPSI-R, DM/PR	Comm meeting; Yes	Cognitive: CC = COMP	ND
							PR Baseline CC < COMP	
							PR 11-mo follow-up: CC = COMP	
Lordelo et al. (2002) [38], Brazil	Adult-child verbal/non-verbal interactions	X-sect	12–42 mo	148 (39.0)	Free-play observation	CC/NR; NR	Nonverbal interactions: home > CC, public CC > private CC	ND
							Verbal interactions: home = CC, public CC = private CC	
Ricardo et al. (2019) [39], Brazil	Physical activity	X-sect	12 mo	2974 (NR)	Accel	2015 PBC; NR	Accelerometry: public/private CC < COMP	NEG

Accel – accelerometer, Anthro – anthropometry, BF – breastfeeding, BMI – body mass index, CC – childcare centre, Comm – community, COMP – comparison group, Dia – diarrhoea, diff – difference, DM – direct measure, EBF – exclusive breastfeeding, ECLS - Early-Childhood Long Survey, HC – health centre, Hosp – hospital, Long – longitudinal, LRI – lower respiratory tract infection, Mo – months, MV – medically verified, ND – no difference, NEG – negative, Neuro-dev – neurodevelopment, NGO – non-governmental organisation, NR – not reported, NS – non-specific, NSHN - National Survey on Health and Nutrition, NUHDSS - Nairobi Urban Health and Demographic Surveillance System, OW – overweight, PBC - Pelotas Birth Cohort, Pneum – pneumonia, POS – positive, PR – parent reported, QOL – Quality of Life, Qual – qualitative, TR – teacher reported, URI – upper respiratory tract infection, UW – underweight, Vax – vaccination, X-sect – cross-sectional, Y – year

*Clark et al. [22] listed twice due to having child health and development outcomes. Oliveira et al. [21] listed twice due to having respiratory and gastrointestinal health outcomes.

**Figure 2:**
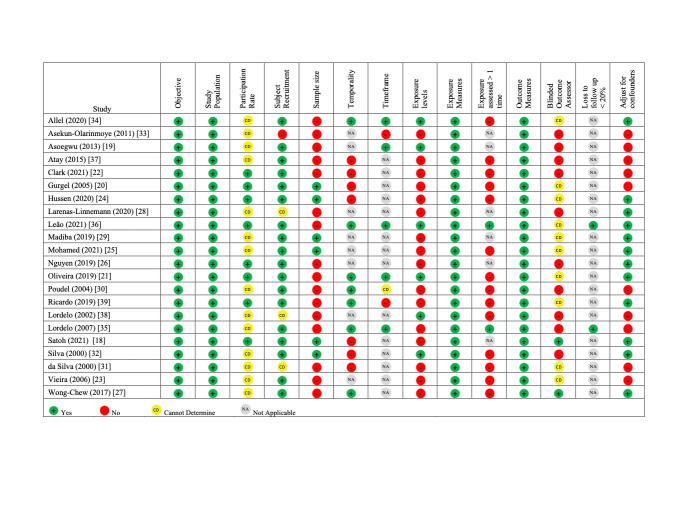
Methodological quality assessment of the included articles (n = 22) using the using NIH National Heart, Lung, and Blood Institute Study Quality Assessment Tool for Observational Cohort and Cross-Sectional Studies [13].

The overall quality of the articles was low (**Figure 2**). Only 41% (n/N = 9/22) met more than half of the quality criteria, 27% (n/N = 6/22) examined levels of childcare exposure (e.g. duration and age of entry), and 45% (n/N = 10/22) adjusted for confounders. Other areas of low quality or missing information included sample size justifications, temporality, assessment of the exposure more than once, blinding the outcome assessor, and participation rate.

### Child health

Twelve studies examined the association between childcare attendance and children’s health.

#### Otitis media with effusion (OME)

Two studies examined the association between childcare attendance and OME using otoscopic evaluations [18,19]. One included 274 children from community health centres in Vietnam [18]. An adjusted analysis found that childcare attendance was associated with greater odds of OME. The other study, from Nigeria, included 152 children from childcare centres and a comparison group from immunisation clinics [19]. The childcare group was substantially older than the comparison (approximately 63% aged 19–24 months vs approximately 90% aged 1–6 months, respectively). An unadjusted analysis found no difference in OME prevalence between the two groups. OME in the childcare group was associated with early enrolment age and longer duration of attendance.

#### Gastrointestinal health

Three studies examined the association between childcare attendance and gastrointestinal health [20–22]. One included 468 children from Aracaju, Brazil recruited children from 10 childcare centres and neighbourhood comparison groups [20]. In unadjusted analyses, the prevalence of intestinal parasitosis was significantly higher among the childcare group compared to the comparison group. The groups differed by environmental conditions and socioeconomic status, with the childcare group from communities having poorer housing conditions (e.g. unpaved streets, no sewage system, garbage collection) and parents with lower educational and income levels. The childcare centres varied in hygienic conditions, food handling and handwashing practices, and child-to-supervisor ratio.

Another Brazilian study examined the association between childcare attendance and infectious diseases among 4018 children from the 12-month follow-up of the 2015 Pelotas Birth Cohort [21]. Most children had not attended childcare (88.3%, n = 3546); 8.7% (n = 351) attended private centres; and 3.0% (n = 121) attended public centres. In adjusted analyses, childcare attendance was associated with greater occurrence of diarrhoea over the previous two weeks, particularly among children who entered childcare recently (after eight months of age). Findings differed when the private and public centres were considered separately. After adjustment, childcare attendance was associated with diarrhoea among private childcare centres, but not among public childcare centres.

The third study included 1337 mothers who participated in the Nairobi Urban Health and Demographic Surveillance System [22]. The childcare group had significantly lower incidence of diarrhoea over the past two weeks than the comparison group. Among employed mothers, childcare attendees’ overall health was rated significantly higher compared to non-attendees. A subset of 31 mothers who participated in a small qualitative sub-study voiced concerns regarding poor sanitary conditions and viruses in childcare centres.

#### Respiratory

Seven studies examined the association between childcare attendance and factors related to respiratory illnesses [21,23–28]. Three found childcare attendance to be associated with bacterial infections and respiratory illnesses [23–25]. One study from Brazil included 200 children from childcare centres or an outpatient clinic in Sao Paulo and in Porto Velho [23]. The prevalence of *Streptococcus pyogenes* was higher among childcare attenders compared to controls in both cities, significantly higher in Sao Paulo, but not in Porto Velho. Although households in Porto Velho had poorer hygienic conditions compared to Sao Paulo, analyses were not adjusted. A second study included 413 children from outpatient clinics in Ethiopia [24], and a third enrolled 698 children using community registration records from six communes in central Vietnam [25]. In adjusted analyses, relative to the comparison group, childcare attendance was associated with significantly higher prevalence of *Streptococcus* pneumonia in the nasopharyngeal carriage [24] and in the conjunctival flora [25], respectively.

Two studies examined the association between childcare attendance and pneumonia [26,27]. A study from Vietnam among 4206 children hospitalized with pneumonia found that in an adjusted analysis, childcare attendance was protective against adverse outcomes [26]. Conversely, an unadjusted study from 11 hospitals in Mexico among 1404 children with community-acquired pneumonia found that childcare attendance was a significant risk factor for severe pneumonia [27].

Two studies examined associations between childcare attendance and respiratory conditions at 12 months of age [21,28]. One Mexican study included 703 children from primary health clinics and found that childcare attendance was not associated with atopic or non-atopic wheezing [28]. Another study on 4018 children from the 2015 Pelotas Birth Cohort in Brazil found through adjusted analyses that childcare attendance was positively associated with non-specific respiratory symptoms, upper- and lower-respiratory infections, but not flu/cold over the previous two weeks [21]. Associations for respiratory infections were stronger for children who entered childcare at an age closer to the outcome evaluation (e.g. entered after eight months of age).

In summary, associations between childcare attendance and outcome measures were primarily negative for health (n/N = 7/12). Three studies found no differences based on childcare attendance and two showed protective associations. Many of the studies did not adjust for confounders (n/N = 6/12) or consider home or childcare factors that could relate to children’s health, such as children’s age, childcare centre’s hygienic practices, or families’ socioeconomic status.

### Child growth and nutrition

Six studies examined the association between childcare attendance and children’s growth or nutrition [29–34].

#### Growth

Four cross-sectional studies used direct measures of heights and weights and the World Health Organization (WHO) child growth standards (z-scores) to define stunting (height-for-age z lower than −2), underweight (weight-for-age z lower than −2), and wasting (weight-for-height z lower than −2). A South African study included 1254 children from well child clinics; in an adjusted analysis, the childcare group was less likely to be underweight or stunted than the comparison group, with no association with wasting [29].

A study from Nepal (n = 46) was conducted in two centres from urban slums that provided supplementary feeding, with a neighbourhood comparison [30]. Stunting rates were lower in the non-governmental organisation centre compared to the comparison group, while stunting and underweight rates did not differ between the municipal centre and comparison group. The hygienic conditions of the municipal centre were poor (i.e. no toilets or water supply). A study based on the 1989 National Survey on Health and Nutrition (n = 10 667 children under six years) across five large regions of Brazil found a higher prevalence of stunting in the comparison group compared to the childcare group [31]. Childcare centre attendance increased with increasing household income. Another study (n = 2122) from an impoverished area of Sau Paulo, compared four municipal childcare attendees to non-attendees [32]. Rates of mild malnutrition were lower among childcare attendees compared to non-attendees. Among attendees, in adjusted analyses, rates of mild malnutrition were lower for children who attended more than one year.

A fifth study from Chile used a longitudinal nationally representative survey (n = 1273) to examine the association between childcare centre attendance and body mass index (BMI) following exclusive maternal care from 12 to 24 months [34]. At 36–48 months, childcare attendance was associated with lower BMI than home-care, with no difference in overweight status and no difference between public and private childcare centres and smaller BMI change for children of lower socioeconomic status.

#### Breastfeeding

One study compared exclusive breastfeeding and breastfeeding duration among 500 children in childcare vs home-care among lower-middle income families in Nigeria [33]. In an unadjusted analysis, the prevalence of breastfeeding exclusivity and duration was lower among childcare attendees, and their mothers were more likely to have completed secondary education and be in professional positions (62%) compared to those of non-attendees.

In summary, the five studies that examined associations between childcare attendance and growth reported positive associations, including four that focused on cross-sectional measures of stunting or underweight and one that focused on longitudinal changes in BMI and overweight. The single study on breastfeeding found lower rates of breastfeeding among childcare attendees compared to non-attendees.

### Child development

Six studies examined the association between childcare attendance and children’s development [22,35–39].

#### Neurodevelopment

Four studies used standardised assessments of neurodevelopment [22,35–37]. One included 37 children from a low-income community in Brazil, either in childcare or in home care [35]. The Bayley Scales of Infant Development Version II (BSID-II) and the Wechsler Preschool and Primary Scale of Intelligence-Revised (WPPSI-R) were administered at enrolment and three follow-ups. In unadjusted analyses, there were no differences between childcare and comparison group scores. A second study included 3870 children from the 2015 Pelotas Birth Cohort in Brazil [36]. The INTERGROWTH-21st Neurodevelopment Assessment was administered at age two. In adjusted analysis, the childcare group had significantly higher scores than the comparison group. A third study administered the Ages and Stages III to parents who participated in the Nairobi Urban Health and Demographic Surveillance System, representing mixed, but primarily low-income communities [22]. The childcare group was significantly less likely to be cognitively delayed than the comparison group. An in-depth qualitative sub-study among 31 mothers reported strong beliefs that childcare benefitted children’s cognitive, social, and emotional development, while ensuring their safety and promoting their social skills and maturity [22]. Maternal concerns included childcare affordability and quality. A study from Turkey administered the Pediatric Quality of Life Inventory to 168 parents from a hospital-based outpatient clinic who varied by childcare attendance [37]. In unadjusted analyses, the childcare group had significantly higher scores overall and on all functional areas (psychosocial health, physical health, social functioning, and emotional functioning) than the comparison group. Mothers of childcare attendees had higher average age, employment prevalence, and school completion, compared to mothers of non-attendees.

#### Adult-child interactions

One study from Brazil observed free play adult-child interactions among 148 children (58 in childcare centres and 90 in low-income households) [38]. In unadjusted analyses, public childcare centre children had significantly more non-verbal interactions than private childcare centre children, with no significant difference in number of verbal interactions. Non-verbal interactions were greater with mothers than with childcare providers, with no difference in verbal interactions.

#### Physical activity

The final study addressed children’s physical activity; 2974 children in the 2015 Pelotas, Brazil birth cohort wore a wrist-attached accelerometer for four days at 12-month [39]. In adjusted analyses, childcare non-attendance was positively associated with measured physical activity, regardless of children’s ability to walk.

In summary, associations between childcare attendance and children’s developmental performance on standardised measures were generally positive (n/N = 3/4). The single study that assessed verbal and non-verbal teacher-child and parent-child interactions had mixed findings. Finally, the single study that assessed associations between childcare attendance and physical activity found less physical activity among attendees compared to non-attendees.

### Childcare centre programmatic and research recommendations

Regarding childcare programming, the review yielded limited information regarding the contextual aspects of the childcare settings and the socioeconomic background of the families. Indicators that suggest low quality programming for children under three years of age were poor hygienic practices; staff-child ratios below recommendations for children aged 0–3 years [3,14]; little mention of provisions for infants (including breastfeeding accommodations); and few comments on curricula, enrichment opportunities, or parent engagement. There was also limited information on childcare affordability and costs, on funding, or on organisational structure (e.g. private, public, and nongovernmental). Children under three years of age require high quality specialised care, including consistent caregiving staff with high staff-to-child ratios and close attention to children’s health, nutrition, and developmental needs, often resulting in high costs [3,4,14].

We organised our programmatic recommendations by extending the criteria proposed by Zero-To-Three [14] to address specific policies for children aged 0–3 years in childcare centres in LMICs, and incorporated the findings related to programmatic quality and study quality from the review. We were also informed by publications that address global childcare quality and policies [1,15,16], as well as the health, nutrition, and developmental needs of infants and young children [3,5,17]. We generated five programmatic recommendations and three research recommendations (**Table 2** and **Table 3**).

**Table 2:** Childcare centre programmatic recommendations for children aged 0–3 years

Programmatic component	Background	Recommendations
Policies for children aged 0–3 y	Policies establish the criteria for safety and high-quality childcare practices, including staff training and support, hygienic conditions, and daily routines. The Nurturing Care Framework [6] calls for multisector policies that address children’s needs across health, growth, and development, particularly during the rapid developmental periods of infancy and young childhood. As of December 2019, 76 countries (39% of 197 countries world-wide) had adopted multisector policies related to early childhood development [16].	Multi-sector policies for children aged 0–3 y can establish the guidelines for nurturing care, including accommodations for breastfeeding, opportunities for attachment with consistent caregivers, and protection from infections and the potential stress of busy childcare centres [3,14].
Programmatic quality of childcare	Programmatic quality of childcare has been a determining factor on child outcomes [68]. Yet, programmatic quality often receives minimal attention.	Monitoring and maintaining programmatic quality includes safety, hygiene, adequate nutrition, and curricula that are culturally- and developmentally-appropriate, and enhance child health, growth, and development.
*Safety*	Young children attending childcare often spend many waking hours in these settings, which may represent their only opportunity to engage in active play. Safety for children under age three years in childcare settings involves protecting them from falls, burns, ingestions, choking, drownings, and other potential hazards [14]. Opportunities to engage in active play contribute to brain development and lay the foundation for further learning in later childhood [6].	Childcare environments without physical dangers or environmental risks, and where young children can safely sleep, eat, and engage in active play under supervision to ensure that their curiosity does not expose them to dangers, will help promote healthy growth and development and build healthy habits [69].
*Hygiene*	Young children’s hygienic practices are emerging. They require diaper changing, hand washing, and protection from frequently putting things into their mouth.	A review of childcare guidelines to reduce infections in childcare centres reported that hand hygiene, cleaning of the environment, and exclusion of children with a fever or illness symptoms were uniformly recommended [41].
*Adequate nutrition*	Breastfeeding is recommended by major health organizations [56,57]. However, mothers of children attending childcare centres face barriers in continuing breastfeeding.	Adherence to global policies for breastfeeding, complementary feeding, and Infant and Young Child Feeding practices advances children’s health, growth, and development [56,57]. Breastfeeding policies include professional development training on supporting breastfeeding in childcare programmes, proper storage and handling guidelines, feeding plans, and a culturally appropriate breastfeeding-friendly environment.
*Culturally- and developmentally-appropriate curricula and activities*	Infants and toddlers have highly specialized needs related to feeding, sleeping, toileting, learning, and physical and social development and require care from a consistent caregiver [69]. Although children’s developmental progression is generally consistent across cultures, cultural variability in caregiving practices enables young children to build culturally relevant habits and expectations.	Children benefit from nurturing interactions from consistent caregivers and curricula that reflects both the expected developmental progression and the culture-specific practices.
Access and affordability	Families need childcare centres that are accessible (close proximity to home and/or workplace) and affordable.	Having available information on the types of childcare centres that serve infants and toddlers (e.g. public, nongovernmental, and private), along with information on subsidies and fees enables families to plan and make appropriate choices for childcare.
Parent engagement	Communicating between parents and childcare staff enables them to share information about children's daily activities and behaviour.	Engage parents through communication and childcare events to facilitate harmony and consistency in meeting children’s needs in childcare and at home.
Financial support and sustainability	Childcare for young children often requires external investment due to the high staff-child ratio required. A simulation study in the UK found that investing in free universal early childcare programmes would lead to short-term employment benefits and reductions in gender inequalities in earnings and long-term benefits in children’s well-being, social and cognitive development, and in their economic productivity through better education, social skills and greater adaptability [70].	Advocate for investment in high quality, affordable childcare for children aged 0–3 y. Identify external funders and investment schemes, as costs of childcare for children aged 0–3 y are high and may be unaffordable for many families [67].
Workforce development	Workforce qualifications vary across countries and types of centres and could relate to children’s health and development and to family satisfaction. Traditionally childcare workers have been paid low wages, often leading to high turnover and the absence of the consistent caregiving that young children need.	Childcare providers need specific training in care for very young children, along with supervision, support, and adequate wages.

**Table 3:** Childcare centre research recommendations for children aged 0–3 years

Research component	Background	Recommendations
Study design and measurement	Many childcare studies are at risk of biased results from not describing characteristics related to children’s exposure to the childcare centre, characteristics of the childcare centre, the sociodemographic and cultural environments, and measuring outcomes for the mother and family.	To reduce the risk of bias in childcare studies, include: child age at enrolment, frequency of attendance, and duration of attendance; type of childcare centre; home and childcare centre sociodemographic and cultural environment; outcomes for children, mothers, families, and childcare staff; approaches to reduce selection bias.
*Child age at enrolment, frequency, and duration*	Variability in child outcome measures may occur, based on exposure to childcare [65], which is often not reported.	Measure age of enrolment, frequency of attendance (e.g. full or part-time), and duration of attendance.
*Type of childcare centre*	In addition to public, government-sponsored childcare centres, childcare centres may be sponsored by nongovernmental organizations, employers, private individuals or companies. Subsidies and payment schedules vary by the type of centre and child age (e.g. higher for infants). Quality may vary by centre type.	Examine differences by centre type, as shown in Lordelo (Brazil) [35] and Poudel (Nepal) [30], and address access, availability, affordability, and quality.
*Home and childcare centre sociodemographic and cultural environment*	Children’s early development is influenced by their sociodemographic and cultural environment in the home and childcare centre. Sociodemographic and cultural environment in the home and childcare centre provides information on the equivalence of comparison groups.	Measure the sociodemographic and cultural environment of the homes and childcare centres (or alternative care sites).
*Outcomes for children, mothers, families, and childcare staff*	In addition to children’s health, growth, and development, childcare attendance can impact mothers, families, and childcare staff [48].	Measure the impact of childcare on children’s families, especially mothers, and on childcare staff, including their workforce participation, income generation, empowerment, and mental health [15].
*Approaches to reduce selection bias*	A key challenge in studying the effect of childcare is identifying an adequate counterfactual given families’ self-selection into childcare centres.	To mitigate selection bias, consider using instrumental variables (i.e. waitlists and lotteries) to randomize allocation, regression discontinuity, and stepped wedge designs [71,72], depending on the context and research objective. Conduct formative research of the childcare landscape to understand childcare attendance alternatives. Reduce selection bias by adjusting for factors that influence enrolment or through causal inference methods such as doubly robust estimation [72]. Use these strategies to study how different childcare environments or family backgrounds relate to the impact of childcare centre attendance on children’s health, growth, and development.
Longitudinal studies	Little is known about the long-term impact of childcare attendance among very young children.	Conduct rigorous longitudinal studies to evaluate the impact of childcare among children under age three years on their health, growth, academic performance, and behavioural development through childhood and adolescence, and economic productivity and well-being in adulthood [15].
Implementation research	Implementation research is needed to understand the components of childcare centre programs that are effective in achieving positive outcomes for young children at greatest risk for poor health, growth, and development.	Using implementation research, conduct systematic studies of methods that support nurturing care for young children in childcare settings with the goal of identifying strategies to implement policies, standards for best practices, and systems for quality improvement that relate positively to young children’s health, growth, and development [73].

The childcare centre programmatic recommendations for children aged 0–3 years targeted policies and programming to ensure safety, high quality, and sustainability. The recommendations included: age-specific policies; programme quality (including safety, hygiene, nutrition, and curriculum); access and affordability; parent engagement; financial support; and workforce development. The research recommendations emphasise the need for ongoing primary research as the number of childcare centres increase in response to women’s increasing role in the workforce and the recognition that children benefit from nurturing care. Research recommendations included: study design (including enrolment age, frequency, duration, childcare type, home and childcare sociodemographic and cultural environments, child and caregiver outcomes, and analytical approaches); longitudinal studies; and implementation research.

## DISCUSSION

With women’s increasing workforce participation and the recognition that young children require nurturing care to meet their developmental potential, childcare for children under three years old has become a global concern [1,5,6]. Our review has three important findings related to this topic. First, childcare studies among children under three years of age are being conducted in LMICs throughout the world, with patterns in children’s health, growth, and development remaining in line with findings from high income countries (HICs). For the most part, early childcare is positively associated with children’s growth and development, and negatively associated with health through an increased risk of infections. Second, as reported in HICs, the programmatic quality of childcare centres for children under the age of three is critically important, ranging from concerns related to safety and hygienic facilities, to staff-child ratios, and developmentally and culturally appropriate curricula for very young children. Low programmatic quality potentially threatens the well-being of young children and may prevent parents from utilising childcare, thus impacting mothers’ workforce participation and productivity. Guidelines for programmatic recommendation for childcare for children under three years of age are needed urgently. Finally, we found relatively low study quality and high risk of bias that could compromise the interpretation of the data. This highlights the need for increased attention to methodological rigour in studying childcare in LMICs, including contextual variables and adjusting for confounders, rather than merely relying on comparisons between childcare attendance and home or alternative care.

Findings from HICs have documented an increased incidence of diarrhoea, otitis media, and respiratory illnesses associated with childcare attendance, particularly among the youngest children [40]. Although most studies found negative associations between attendance and health, there were also conflicting findings, potentially due to the lack of information on home and childcare contextual factors, thus prohibiting adjusted analyses to control for contextual differences. Evidence suggests that some children acquire immunity and resilience over time [41].

In response to the threat of infections, childcare centres have implemented and evaluated hygienic policies and practices through handwashing and hand sanitizers, isolating ill children, or restricting attendance until the illness has passed. Although these methods are effective in reducing infections [42,43], barriers include the availability of soap and water, hand sanitizer, infrastructure, adherence [44,45], and insufficient time for children to adopt newly introduced hygiene practices [43,46,47].

We found that childcare centres can support child nutrition, particularly in the first three years when growth is rapid. Consistent with the longitudinal study in Chile [34], a study in Brazil among children aged 0–3 years from low-income homes provided five meals or snacks daily and found that children had healthy height and weight gains that were sustained over seven years [48]. In HICs, associations between childcare attendance and children’s nutrition and growth are limited, particularly for children under three years of age [8]. One exception is a Danish study that found that childcare attendance in the first year of life was associated with slightly higher BMI z-scores and increased odds of overweight or obesity at 12 months of age [49]. Systematic reviews of childcare attendance among children under six years of age have reported mixed findings related to obesity risk, largely associated with variability in age of enrolment, centre nutrition, and physical activity environment [50,51].

In HICs, childcare attendance has been associated with shorter breastfeeding duration [52] consistent with the study in Nigeria [33]. Childcare centres can support breastfeeding by providing opportunities to breastfeed on-site, training staff on breastmilk handling, storage practices, providing cold storage, and bottle and cup hygiene [53]. Despite the well documented benefits of breastfeeding for children’s health and immune function [54,55], policies related to breastfeeding and breastmilk provision in childcare centres are lacking globally [1]. Breastfeeding accommodations and attention to young children’s nutritional and feeding needs [56,57] are especially important given the increased risk of illness associated with childcare attendance.

Evidence from HICs has shown that childcare centres can contribute to young children’s early development by adhering to high standards of programmatic quality, including staff-child ratio and indoor and outdoor space [58]. Additional indicators of high quality include staff who are well-trained, supervised, and supported; groups size that facilitate responsive adult-child interaction; developmentally and culturally-appropriate curricula with learning opportunities; and settings that are safe, sanitary, and accessible to parents [15,40,59]. Childcare-related advances in language and socioemotional skills are often strongest among children from disadvantaged or immigrant families, with reductions in disparities among children enrolled early in life [60].

Childcare studies among children under age three years among Organisation for Economic Co-operation and Development (OECD) and other HICs have had mixed findings related to child development [40]. Children of mothers with limited education may differentially benefit in language and cognitive development, particularly when childcare centres provide nurturing, stimulating, and age-appropriate opportunities [40,61]. For children who have experienced limited opportunities for responsive play and learning opportunities, interpersonal interactions in childcare may be particularly beneficial, especially when childcare is initiated during infancy [62]. Children’s childcare experiences are highly contingent on the quality of the childcare, such that community economic restraints and lack of childcare provider training and support can result in negative effects on children’s development [15].

The variability in the association between childcare attendance and physical activity [39] has been demonstrated previously [63], primarily due to the high prevalence of sedentary activities in childcare settings. Recommendations for increased emphasis on physical activities in childcare centres includes space and curricula adjustments emphasising motor development [64].

None of the reviewed studies measured the children’s behaviour. This lack of attention to behaviour is a concern, particularly because several studies have reported negative behaviour (e.g. aggression) associated with childcare attendance, accentuated in the context of low childcare quality or extended duration [65]. Stress-related behavioural problems may occur, particularly among children with difficulties interacting with peers or insensitive parents [40].

Adult-child interactions are rarely considered in childcare studies, yet children build cognitive and socio-emotional skills through interpersonal interactions, emphasising the importance of attention to the quality and quantity of interactions. The absence of differences in adult-child verbal interactions between childcare and home-care suggests that childcare settings may provide opportunities for children to engage in responsive and stimulating adult-child interactions [38].

The strengths of our review include an exclusive focus on childcare centres in LMICs that serve children aged 0–3 years with wide global representation, as well as a systematic review of the study quality through independent ratings by two reviewers, with a third independent rating for consensus. Consistent with children’s need for nurturing care, we examined multiple outcomes to obtain a holistic view of children’s health, growth, and development. Finally, we reviewed the programmatic quality, as reported in the studies. Using information from the reviews, principles of nurturing care, and caregiving requirements for infants and toddlers [6], we generated five programmatic recommendations and three research recommendations focused on children under age three years – a topic that has been understudied and is of urgent need.

Besides the methodological limitations noted in the reviewed articles, we may have missed articles based on several decisions. We limited the review to childcare centre comparisons, excluding family childcare homes or other informal care. We also restricted the search to articles published in peer-reviewed journals either in English or with English translation, meaning that we may have missed articles from non-governmental organisations or published in languages other than English. We did not expand our search to additional databases (i.e. Web of Science and Scopus) and due to coronavirus disease (COVID-19) related closures and delays, our search did not extend beyond 2021.

## CONCLUSIONS

Overall, we found generally positive associations between childcare attendance among children under three years of age in LMICs and young children’s growth and development and negative associations with their health. These findings are largely consistent with childcare studies conducted in HICs among the same population. The variability in findings is at least partially attributed to low study quality, low programmatic quality, and missing information on family and childcare context, childcare type, and other aspects of childcare centre operations. Despite the positive associations with growth and development, childcare may pose a risk to young children through reductions in breastfeeding exclusivity and duration, increased infections, and a potential lack of opportunities for learning and responsive interactions if programmatic quality is low. Children exposed to adverse home conditions appear to differentially benefit from childcare programmes guided by nurturing care, particularly centres that are well-organized to provide the hygienic care, nutrition, feeding environment, and interpersonal play and learning opportunities that are needed by young children [40].

Lack of childcare is frequently cited as the primary barrier to maternal employment [66]. The lack of affordable and quality childcare restricts women’s employment opportunities and productivity [1,67]. Balancing parental leave, childcare, and women’s role in the workforce can be challenging. Having reliable, high-quality childcare can enable women to advance their employment opportunities and their empowerment in decision-making and management. Very little research has extended the evaluation of childcare beyond children to include mothers and other family members.

Finally, many LMICs are increasing their attention and support for early childcare in support of maternal employment and young children’s need for nurturing care. With the recognition that lifelong health, well-being, and productivity are influenced by nurturing environments for children under age three years [5,6], childcare programmes provide an opportunity for countries to overcome challenges associated with early adversities, such as undernutrition, and resource-limited communities and homes. Policies, programmes, and investments that support high-quality childcare can support women in the workforce and enhance country-level progress by ensuring that young children receive nurturing care needed to protect and advance their health, growth, and development. Investing in early childcare can lay the foundation for a brighter future for children, for women, for families, and for countries.
